# Factors associated with changing indications for adenotonsillectomy: A population-based longitudinal study

**DOI:** 10.1371/journal.pone.0193317

**Published:** 2018-05-29

**Authors:** Jeng-Wen Chen, Po-Wu Liao, Chi-Jeng Hsieh, Chu-Chieh Chen, Shang-Jyh Chiou

**Affiliations:** 1 Department of Otolaryngology-Head and Neck Surgery, Catholic Cardinal Tien Hospital and School of Medicine, Fu-Jen Catholic University, New Taipei City, Taiwan; 2 Department of Otolaryngology-Head and Neck Surgery, National Taiwan University Hospital, Taipei, Taiwan; 3 Department of Otolaryngology, Catholic Yonghe Cardinal Tien Hospital, New Taipei City, Taiwan; 4 Department of Health Care Administration, Oriental Institute of Technology, New Taipei City, Taiwan; 5 Department of Health Care Management, National Taipei University of Nursing and Health Sciences, Taipei, Taiwan; Technische Universitat Munchen, GERMANY

## Abstract

**Objective:**

Adenotonsillectomy (AT) is one of the most common surgical procedures performed in children and adults. We aim to assess the factors associated with changes in the incidence of and indications for AT using population-level data.

**Study design:**

This retrospective cohort study investigated patients who underwent AT between 1997 and 2010 by using data from the Taiwan National Health Insurance Research Database. We examined surgical rates and indications by the calendar year as well as age, sex, hospital level, and insured residence areas for the correlating factors.

**Results:**

The average annual incidence rate of AT was 14.7 per 100,000 individuals during 1997–2010. Pediatric (<18 years) patients represented 48.2% of the total AT population. More than 99% of the patients underwent the AT procedures as an inpatient intervention. Longitudinal data demonstrated an increasing trend in the pediatric AT rates from 1997 (4.3/100,000) to 2010 (5.7/100,000) (*p* = 0.029). In the adult subgroup, a decreasing prevalence of infectious indications (*p* = 0.014) coincided with an increasing neoplastic indications (*p* = 0.001). In the pediatric subgroup, the prevalence of obstructive indications increased (*p* = 0.002). The logistic regression analyses indicated that the significant factors associated with the changing surgical indications for AT were the age in the adult subgroup and hospital level in the pediatric subgroup.

**Conclusions:**

This study revealed a low AT rate in Taiwan than that in other countries. Pediatric AT incidence increased during 1997–2010. Although a rising prevalence of obstructive and neoplastic indications was noted, infection remained the most common indications for AT. Age in the adult subgroup and hospital level in the pediatric subgroup were factors associated with the changing indications for AT.

## Introduction

Tonsil surgery has been performed for more than 3,000 years [1, 2]. Historically, tonsillectomy was mainly performed to treat the infectious complications of tonsillitis [2, 3]. With the introduction of oral antibiotics in the 1960s, the number of tonsillectomies declined; however, it is still one of the most common surgical procedures performed in Western countries [3–7]. Adenoidectomy is a regular surgical procedure performed by otolaryngologists. Occasionally, an adenoidectomy is performed concurrently with tonsillectomy. An adenoidectomy performed alone or with other procedures such as tympanostomy tube placement, or nasal surgery may exhibit different characteristics. The variation in adenotonsillectomy (AT) rates across different countries may be explained in part by the different cultural attitudes toward the protective role of these lymphoepithelial structures [8, 9], use of antibiotics for throat infections, and absence of generally accepted indication criteria for surgery [10, 11]. A 22-year nationwide cohort showed a significant decrease in the incidence of tonsillectomy over time [12]; however, regional variations were observed in the number of tonsillectomies. Following the introduction of a national clinical guideline for the management of sore throat and indications for tonsillectomy, Mcleod et al. reported a probable association between an increase in admissions with tonsillitis or peritonsillar abscess and a decrease in the rate of tonsillectomy [13]. In contrast to the abundance of information on the incidence trends of AT in Western countries [3–5, 14, 15], limited data exist for Eastern countries [16].

The role of recurrent throat infections as a primary indication for AT is debatable. When the most stringent criteria for AT were applied, a randomized clinical trial [17] showed that surgery was modestly beneficial. However, the same study also provided support for nonsurgical management because during the 3-year follow-up period, many participants in the nonsurgical groups had fewer than three episodes of throat infection, most of which were mild. By contrast, when less frequent or milder illness led to AT, little to no benefit was found [18]. Another study revealed a low level of consensus among pediatricians and otolaryngologists on the appropriateness of AT [11]. Evidence-based guidelines regarding repeated throat infections as an indication for AT are still lacking, which leaves this procedure in the category of “preference-sensitive care” [19]. Differences in clinical practice remain an obvious but inadequately documented problem [20].

The surgical rate of pediatric AT varies notably among different countries [21–23] because of the high accessibility of medical resources when throat infections occur and the cultural acceptability of antibiotic treatment for upper respiratory tract infections. Additionally, discrepancies exist between physicians and parents regarding the use of surgical intervention for recurrent throat infections. Most parents are concerned about the effects of tonsil removal on the immune system and may seek a second opinion regarding surgical options. Furthermore, physicians with different subspecialties may have dissimilar stances on the indications for surgical intervention [11].

In this study, we investigated the factors associated with the incidence and changing indications of tonsillectomy and AT from 1997 to 2010 in Taiwan. These epidemiological data will provide valuable information for the daily practice of physicians, and contribute to the development of an accepted clinical guideline for these procedures.

## Materials and methods

### Study design and database

We conducted a retrospective, population-based cohort study using data from the National Health Insurance Research Database (NHIRD); the NHI commenced in 1995 and covers over 99% of the population in Taiwan [24]. The Longitudinal Health Insurance Database (LHID) contains the complete claims data from 2005 of 1 million beneficiaries. The authors used the registry files from 1997 to 2010 and all applications for reimbursements regarding the inpatient and outpatient healthcare services provided to each patient for analysis. The Institutional Review Board of Cardinal Tien Hospital approved this study (CTH-103-3-5-035) and waived informed consent because the datasets consisted of anonymized, de-identified nationwide data.

### Study population

All patients who had undergone a tonsillectomy without adenoidectomy (International Classification of Diseases, Ninth Revision, Clinical Modification [ICD-9-CM] procedure code 28.2) or tonsillectomy with adenoidectomy (ICD-9-CM procedure code 28.3) between 1997 and 2010 were identified from the representative samples from the NHI program. Patients who underwent an adenoidectomy without tonsillectomy (ICD-9-CM procedure code 28.6) were excluded. All claims data, including demographic administrative and clinical information from both the inpatient and outpatient databases, were used in the analysis. The index date was defined as the time that the tonsillectomy or AT was performed.

### Identification of surgical indications

A list of diagnoses (according to the ICD-9-CM) was obtained from the database for each patient on the index date of surgery. These top three diagnoses were categorized into either infectious or inflammatory indications (recurrent infection or chronic inflammation [RICI]), obstructive indications (tonsillar hypertrophy or upper airway obstruction [UAO]), or neoplastic indications (suspicious benign or malignant neoplasms [Tumor]). If more than one diagnosis fit into a particular category, only the first appearing diagnosis was used to define the indication for AT. The leading ICD-9-CM codes for the indications of RICI, UAO, and Tumor were 474, 780, and 146, respectively. Table 1 shows the complete list of ICD-9-CM codes (top three diagnoses) in these categories of surgical indications.

**Table 1:** The complete list of ICD-9-CM codes in the categories of surgical indications (top three diagnoses).

*Category*	ICD-9-CM codes with descriptions
*RICI*	474 (72%) chronic disease of tonsils and adenoids
	381 (15%) nonsuppurative otitis media and eustachian tube disorders
	683 acute lymphadenitis
	682 cellulitis and abscess of unspecified sites
	478 cellulitis and perichondritis of larynx
	477 allergic rhinitis
	475 peritonsillar abscess
	473 chronic sinusitis
	472 chronic pharyngitis and nasopharyngitis
	463 acute tonsillitis
	462 acute pharyngitis
	461 acute sinusitis
	382 acute suppurative otitis media
	289 other diseases of blood and blood-forming organs
	41 alcoholism in family
	39 liveborn unspecified whether single twin or multiple born in hospital
*UAO*	780.53 (55%) hypersomnia with sleep apnea, unspecified
	786 (15%) symptoms involving respiratory system and other chest symptoms
	756 anomalies of skull and face bones
	750 other congenital anomalies of upper alimentary tract
	749 cleft palate and cleft lip
	519 other diseases of respiratory system
	518 other diseases of lung
	314 hyperkinetic syndrome of childhood
*Tumor*	146 (32%) malignant neoplasm of oropharynx
	196 (18%) secondary and unspecified malignant neoplasm of lymph nodes of head, face, and neck
	210 (11.7%)benign neoplasm of lip, oral cavity, and pharynx
	239 neoplasm of unspecified nature
	141 malignant neoplasm of tongue
	202 other malignant neoplasms of lymphoid and histiocytic tissue
	145 malignant neoplasm of other and unspecified parts of mouth
	200 lymphosarcoma and reticulosarcoma
	147 malignant neoplasm of nasopharynx
	198 secondary malignant neoplasm of other specified sites
	148 malignant neoplasm of hypopharynx
	149 malignant neoplasm of other and ill-defined sites within the lip, oral cavity and pharynx
	161 malignant neoplasm of larynx
	215 other benign neoplasm of connective and other soft tissue
	228 hemangioma and lymphangioma any site
	229 benign neoplasm of unspecified site
	235 neoplasm of uncertain behavior of digestive and respiratory systems
	238 neoplasm of uncertain behavior of other and unspecified sites and tissues
	527 diseases of the salivary glands
	528 diseases of the oral soft tissues excluding lesions specific for gingiva and tongue

ICD-9-CM: International Classification of Diseases, Ninth Revision, Clinical Modification.

RICI: recurrent infection or chronic inflammation.

UAO: tonsillar hypertrophy or upper airway obstruction.

Tumor: suspicious benign or malignant neoplasms.

### Study variables

We investigated the distribution of the three major categories of surgical indication according to sex, age group (<5 years, 5–11 years, 11–17 years, 18–40 years, and >40 years), hospital level (medical centers, regional hospitals, and local hospitals), and insured residence areas according to the NHI divisions (Taipei, Northern, Central, Southern, Kaoping, and Eastern). These variables were considered as the possible factors associated with the incidence of and indications for AT. We presented the data in three different groups, i.e., a total study population, an adult subgroup (≥18 years), and a pediatric subgroup (<18 years) for comparison.

### Statistical analysis

We evaluated the incidence of AT by the calendar year from 1997 to 2010 according to the index date of surgery. We presented the distribution of the three major categories of surgical indication (RICI, UAO, and Tumor) as the number and percentage of patients and analyzed the trends of changing surgical indications. SAS version 9.2 (SAS Institute, Inc., Cary, NC, USA) was used for all the analyses. Descriptive statistics were analyzed using Pearson’s chi-square test. We performed a simple linear regression model to examine the trends in surgical rates and indications by the calendar year. Multinomial logistic regression was established after adjustment for potential confounding effects of age, sex, hospital level, and insured residence areas to identify the possible factors associated with the different surgical indications. The significance was set at a two-sided *p* < 0.05.

## Results

**Fig 1:**
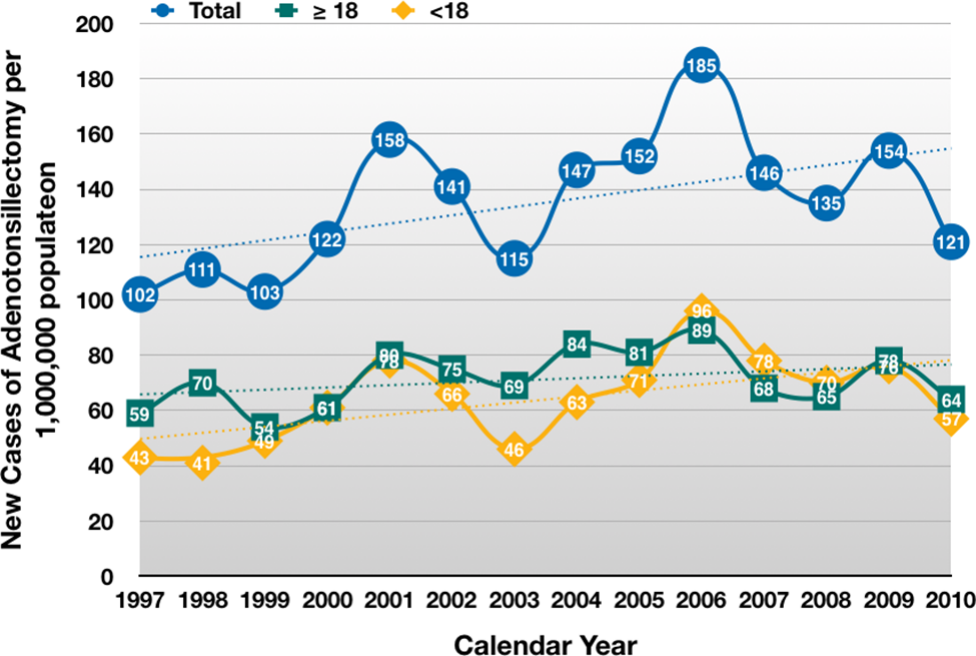
The incidence of adenotonsillectomies per one million population by the calendar year. The adenotonsillectomy rate fluctuates. Linear regression analysis shows an increasing trend in the total study population, in the adult group and in the pediatric group.

Several studies have confirmed the validity and reliability of the NHI LHID from 1 million representative beneficiaries in clinical research [25–27]. A total of 1,892 ATs were performed between 1997 and 2010. The average annual incidence of AT was 14.7 per 100,000 individuals during the study period. The sample comprised 41.3% female patients, with a mean age of 24.0 ± 18.2 years. Patients aged <18 years formed 48.2% of the total AT population. Table 2 shows the number and percentage of patients who received tonsillectomy alone or tonsillectomy with adenoidectomy by age groups in this cohort. Tonsillectomy alone is more commonly performed in adults (920/1195, 76.9%). In contrast, tonsillectomy with an adenoidectomy is most often performed in patients of 5–11 y/o (415/697, 59.5%). Only two patients (2/1892, 0.1%) underwent AT on an outpatient basis. Mean hospital stay of AT patients was 3.7 ± 2.9 days (median, three days). Fig 1 shows the number of ATs by the calendar year. The overall AT rate increased steadily from 102 (per million) in 1997 to 185 (per million) in 2006, with the only exceptions occurring in 2002 and 2003. After 2006, the total number of patients who received an AT decreased sharply to 121 in 2010. However, linear regression analysis shows an overall increasing trend in the total study population (y = 3.0242x − 5923.8, p = 0.052), in the adult subgroup (y = 0.833x – 1597.6, p = 0.235) and in the pediatric subgroup (y = 2.1912x – 4326.2, p = 0.029), respectively.

**Table 2:** Number and percentage of patients who received tonsillectomy alone or tonsillectomy with adenoidectomy by age groups.

	28.2 (tonsillectomy without adenoidectomy)	28.3 (tonsillectomy with adenoidectomy)	Total N (%)
*<5 y/o*	40 (3.3%)	139 (19.9%)	179 (9.5%)
*5–11 y/o*	149 (12.5%)	415 (59.5%)	564 (29.8)
*12–17 y/o*	86 (7.2%)	66 (9.5%)	152 (8.0%)
*18–40 y/o*	524 (43.8%)	60 (8.6%)	584 (30.9%)
*>40 y/o*	396 (33.1%)	17 (2.4%)	413 (21.8%)
*Total N (%)*	1,195 (63.2%)	697 (36.8%)	1,892 (100%)

Data was shown in n (%).

**Fig 2:**
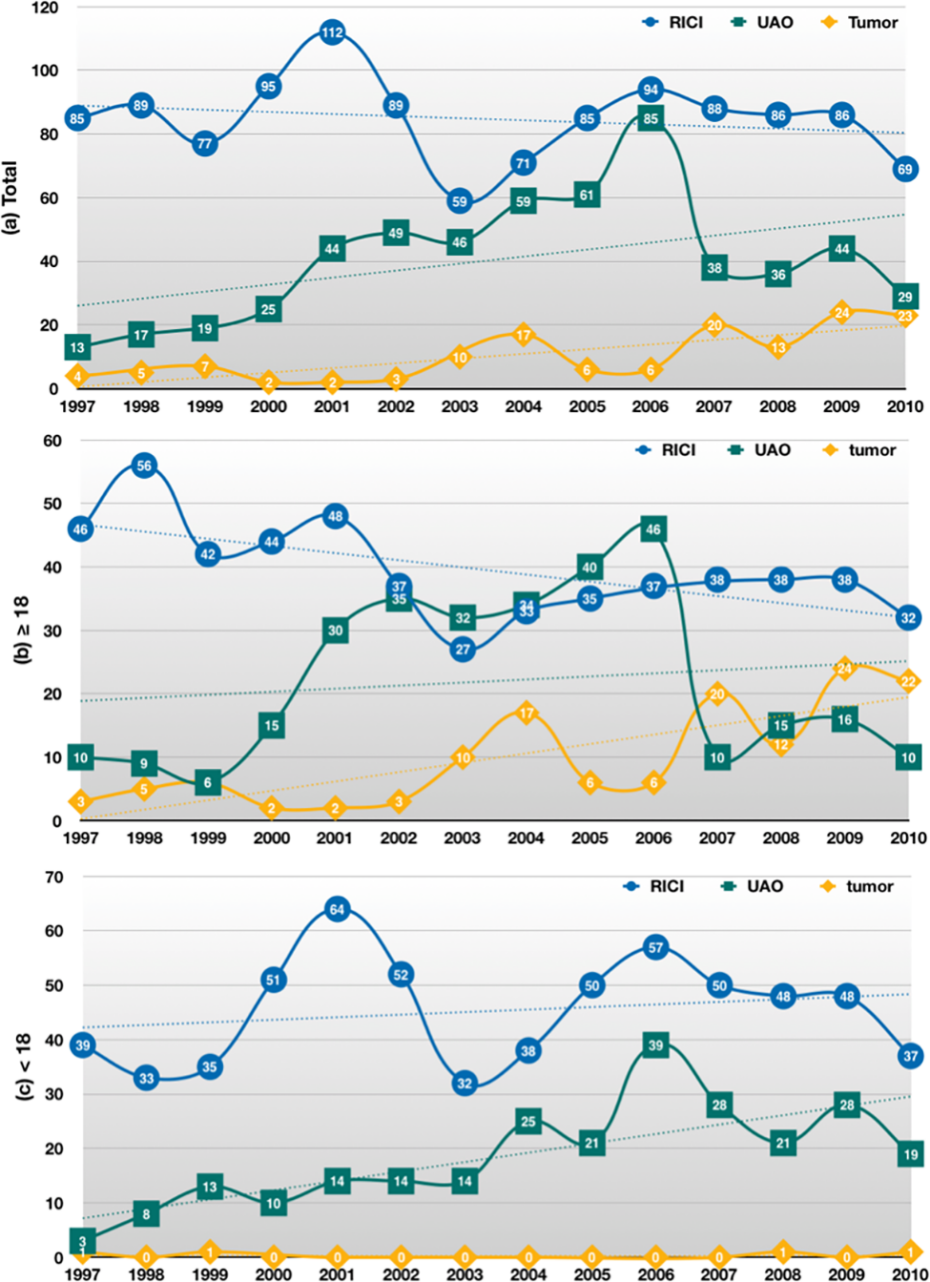
New cases of adenotonsillectomy per one million population of the three categories of surgical indications by the calendar year. 2a: the total study population, 2b: the adult subgroup (≥18 years), 2c: the pediatric subgroup (<18 years). RICI: recurrent infection or chronic inflammation; UAO: tonsillar hypertrophy or upper airway obstruction; Tumor: suspicious benign or malignant neoplasms.

**Fig 3:**
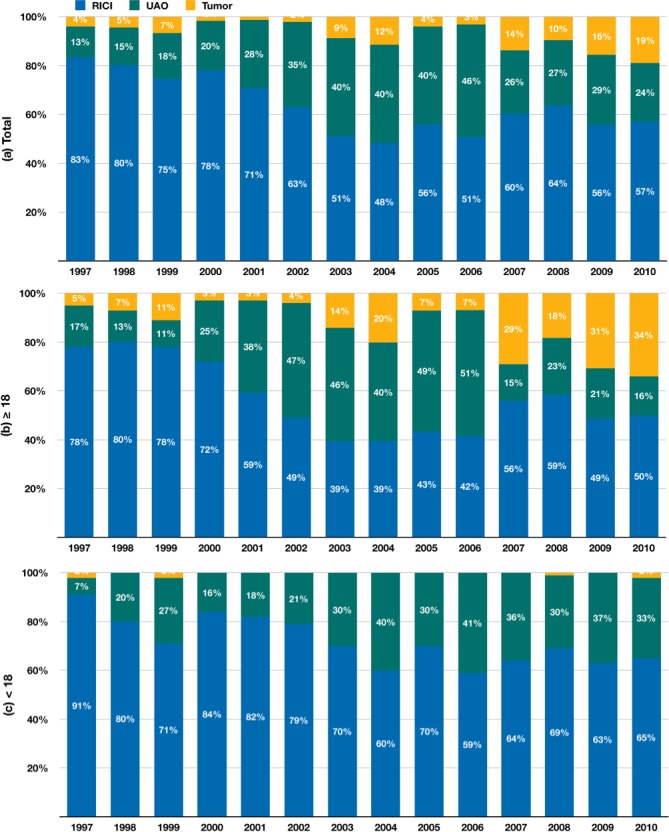
Stacked percentage of three categories of surgical indications for adenotonsillectomy by the calendar year. 3a: the total study population, 3b: the adult subgroup (≥18 years), 3c: the pediatric subgroup (<18 years). RICI: recurrent infection or chronic inflammation; UAO: tonsillar hypertrophy or upper airway obstruction; Tumor: suspicious benign or malignant neoplasms.

Figs 2A and 3A illustrate the incidence and the stacked percentage of the three categories of surgical indications for AT by the calendar year in the total study population. Although the number (blue line) and percentage (blue bar) of RICI declined from 85 (83.3%) in 1997 to 69 (57.0%) in 2010, RICI remained the most prevalent indication for AT, followed by UAO and Tumor. For UAO, both the total number (green line) and percentage (green bar) of patients showed a surge between 2001 and 2006. A significant decrease in the UAO category from 85 (46.0%) in 2006 to 29 (24.0%) in 2010 was demonstrated. Nonetheless, the incidence of UAO showed an increasing trend. The total number of patients and the percentage of Tumor were relatively small from 1997 to 2006, with exceptions in 2003 and 2004, when a transient increase in the incidence and percentage was noted, coincided with a sharp decline in RICI and a modest decrease in UAO. During 2006–2010, the rate and proportion of Tumor increased markedly from 6 (3.2%) in 2006 to 23 (19.0%) in 2010, coincided with the rapid decrease in UAO. Further inspection of the data of these patients revealed that the three leading diagnoses were ICD-9-CM 146 (malignant neoplasm of oropharynx), 196 (secondary and unspecified malignant neoplasm of lymph nodes of head, face, and neck), and 210 (benign neoplasm of lip, oral cavity, and pharynx) at 32%, 18.0%, and 11.7%, respectively. The overall incidence of the Tumor category demonstrated a noticeable increasing trend. Among the total study population, RICI showed a decreasing trend (y = –0.6571x + 89.571, p = 0.462), whereas UAO and Tumor showed an increasing trend (y = 2.2044x + 23.824, p = 0.091 and y = 1.4769x – 0.9341, p = 0.001, respectively).

In the adult subgroup (Figs 2B and 3B), the incidence and proportion of AT performed for RICI decreased from 46 (78%) in 1997 to 32 (50%) in 2010 (y = –1.1275x + 2298.2, p = 0.014), whereas those performed for Tumor increased noticeably from 3 (5%) in 1997 to 22 (34%) in 2010 (y = 1.4769x – 2949.2, p = 0.001). Incidence and percentage of UAO surged between 2001 and 2006 and decreased markedly after 2006, showing a weak increasing trend (y = 0.4835x – 946.73, p = 0.609). By contrast, in the pediatric subgroup (Figs 2C and 3C), indications of RICI and UAO both showed an increasing trend (y = 0.4703x – 897.02, p = 0.486 and y = 1.7209x – 3429.4, p = 0.002, respectively).

Table 3 indicates the number and percentage of the three categories of surgical indications according to sex, age groups, hospital level, and insured residence areas in the total study population. The male patients had a higher overall AT rate and were more likely to undergo AT with the indications of UAO and Tumor, whereas the female patients were more liable to undergo surgery with the indication of RICI. However, RICI remained the most prevalent indication for AT, followed by UAO and Tumor, in both the male and female patients.

**Table 3:** The number and percentage of the three categories of surgical indications according to sex, age groups, hospital level, and insured residence areas in the total study population.

		RICI	UAO	Tumor	*p-*value
**Gender**					
	*Female*	556 (71.2)	183 (23.4)	42 (5.4)	<0.001^a^
	*Male*	629 (56.6)	382 (34.4)	100 (9.0)	
**Age groups**					
	*<5y*	127 (70.9)	51 (28.5)	1 (0.6)	<0.0001^a^
	*5–11y*	395 (70.0)	169 (30.0)	0 (0)	
	*12–17y*	112 (73.7)	37 (24.3)	3 (2)	
	*18–40y*	379 (64.9)	183 (31.3)	22 (3.8)	
	*>40y*	172 (41.6)	125 (30.3)	116 (28.1)	
	*mean ± SD*	20.8 ± 16.5	24.2 ± 17.3	49.6 ± 13.4	<0.0001^b^
**Hospital level**					
	*Medical centers*	628 (61.1)	319 (31.1)	80 (7.8)	<0.0001^a^
	*Regional hospitals*	456 (63.2)	208 (28.8)	58 (8.0)	
	*Local hospitals*	101 (70.6)	38 (26.6)	4 (2.8)	
**Insured residence areas**^**c**^					
	*Taipei D (36*.*6)*^d^	529 (66.1)	207 (25.9)	64 (8.0)	<0.0001^a^
	*Northern D (14*.*0)*^d^	129 (63.2)	58 (28.4)	17 (8.3)	
	*Central D (18*.*4)*^d^	229 (60.1)	132 (34.6)	20 (5.2)	
	*Southern D (13*.*9)*^d^	131 (55.3)	86 (36.3)	20 (8.4)	
	*Kaoping D (14*.*8)*^d^	114 (59.1)	60 (31.1)	19 (9.8)	
	*Eastern D (2*.*3)*^d^	31 (64.6)	15 (31.3)	2 (4.2)	

Data was shown in n (%).

RICI: recurrent infection or chronic inflammation.

UAO: tonsillar hypertrophy or upper airway obstruction.

Tumor: suspicious benign or malignant neoplasms.

D: district.

^a^Pearson’s chi-square test.

^b^Analysis of variance (ANOVA).

^c^missing n = 29.

^d^Percentage of the population in individual NHI division.

The distribution of the surgical indications across each age group differed significantly (*p* <0.0001). Adolescence aged 12–17 years were the most likely to undergo AT because of infectious indications, with 73.7% of these patients undergoing surgery for this reason. Another 24.3% of the patients in this age category underwent surgery due to UAOs. Only 2% of these patients received surgery because of a suspected or confirmed neoplasm. The pattern of the distribution of surgical indications remained similar in the <5 years and 5–11 years age groups. However, the proportion of infectious indications decreased in the adult subgroups. The percentage of UAO was relatively stable across all age groups. Notably, the proportion of Tumor indications increased dramatically in the >40 years age group (28.1%). Compared with the pediatric age groups and 18–40 years group, that of >40 years had more Tumor indications, which coincided with a decrease in the infectious indications.

Most of the ATs were performed in the medical centers and regional hospitals (Table 3). Infection was the most frequent cause of surgery at all hospital levels (61.1% in medical centers, 63.2% in regional hospitals, and 70.6% in local hospitals). The distribution of surgical indications across the hospital level was significantly different (*p* <0.0001). The distribution pattern of surgical indications was similar in medical centers and regional hospitals, those performed in local hospitals were more likely to have been caused by infection and less likely to have been caused by UAO or Tumor. The trend in the AT rate and the proportion of surgical indications by the calendar year according to sex, age groups, hospital level, and insured residence areas in the total study population are shown in the supplemental data (S1 File).

We examined the incidence rate and distribution of the surgical indications for AT among the six distinct insured residence areas in Taiwan (Table 3). Most of the ATs were performed in the Taipei district (42.9%, 800/1863), followed by the Central (20.5%, 381/1863), South (12.7%, 237/1863), Northern (10.9%, 204/1863), Kaoping (10.4%, 193/1863), and Eastern districts (2.6%, 48/1863). The distribution of the surgical indications among the insured residence areas was significantly different (*p* <0.0001). We performed multinomial logistic regression to identify the possible factors associated with the different surgical indications after adjustment for age, sex, hospital level, and insured residence areas, in which UAO was the reference group. We compared RICI with UAO and Tumor with UAO in the same model (Table A in S2 File). After adjusted with other variables, RICI occurred more often in the younger and female patients compared with UAO. Tumor occurred more often in the older patients compared with UAO.

In addition, we examined the related factors (sex, age groups, hospital level, and resident areas) with a time series (S1 File). The patients in the 5–11 years group had a higher proportion of AT due to UAO after 2006, whereas the proportion in the 18–40 and >40 years group declined after 2006. The patients in the 5–11 years of age had a higher incidence of AT because of RICI after 2000, whereas the incidence in the 18–40 years group decreased after 2000. Moreover, the incidence of patients with RICI for AT shifted from medical centers to regional hospitals during the study period, whereas the incidence of surgical indications for UAO and Tumor did not change significantly. Most patients with UAO and Tumor indications received AT surgery at medical centers.

Tables 4 and 5 indicate the number and percentage of the three categories of surgical indications according to sex, age groups, hospital level, and insured residence areas in the adult and pediatric subgroups, respectively. In the adult subgroup, the distribution of the surgical indications in gender (p < 0.001), age (p < 0.0001), hospital level (p < 0.0001) and insured residence areas (p = 0.005) differed significantly. Multinomial logistic regression (Table B in S2 File) showed that age was the only significant factor associated with the different surgical indications in the adult subgroup. Compared with RICI, UAO occurred more frequently in the higher hospital level. The effect of sex, an insured residence area, and hospital level was not statistically significant in the Tumor group (vs. UAO) after all the variables were controlled. By contrast, the distribution of the surgical indications in the pediatric subgroup differed significantly in hospital level (p < 0.0001). Logistic regression (Table C in S2 File) showed that hospital level was the only significant factor associated with the different surgical indications in the pediatric subgroup.

**Table 4:** The number and percentage of the categories of surgical indications according to sex, age, hospital level, and insured residence areas in the adult subgroup (≥18y).

		RICI	UAO	Tumor	*p-*value
**Gender**					
	*Female*	315 (57.2)	98 (31.8)	39 (28.3)	<0.001^a^
	*Male*	236 (42.8)	210 (68.2)	99 (71.7)	
**Age**					
	*mean ± SD*	35.4 ± 13.0	37.6 ±11.9	50.7 ± 12.0	<0.0001^b^
**Hospital level**					
	*Medical centers*	305 (55.4)	159 (51.6)	78 (56.5)	<0.0001^a^
	*Regional hospitals*	187 (33.9)	135 (43.8)	57 (41.3)	
	*Local hospitals*	59 (10.7)	14 (4.5)	3 (2.2)	
**Insured residence areas**^**c**^					
	*Taipei D (36*.*6)*^d^	249 (46.8)	107 (35.3)	62 (44.9)	0.005^a^
	*Northern D (14*.*0)*^d^	64 (12.0)	28 (9.2)	16 (11.6)	
	*Central D (18*.*4)*^d^	86 (16.2)	62 (20.5)	19 (13.8)	
	*Southern D (13*.*9)*^d^	48 (9.0)	54 (17.8)	20 (14.5)	
	*Kaoping D (14*.*8)*^d^	68 (12.8)	45 (14.9)	19 (13.8)	
	*Eastern D (2*.*3)*^d^	17 (3.2)	7 (2.3)	2 (1.4)	

Data was shown in n (%).

RICI: recurrent infection or chronic inflammation.

UAO: tonsillar hypertrophy or upper airway obstruction.

Tumor: suspicious benign or malignant neoplasms.

D: district.

^a^Pearson’s chi-square test.

^b^Analysis of variance (ANOVA).

^c^missing n = 24.

^d^Percentage of the population in individual NHI division.

**Table 5:** The number and percentage of the categories of surgical indications according to sex, age, hospital level, and insured residence areas in the pediatric subgroup (<18y).

		RICI	UAO	*p-*value
**Gender**				
	*Female*	241 (38.0)	85 (33.1)	0.166^a^
	*Male*	393 (62.0)	172 (66.9)	
**Age**				
	*mean ± SD*	8.1 ± 3.9	8.0 ±3.5	0.972^b^
**Hospital level**				
	*Medical centers*	323 (50.9)	160 (62.3)	<0.0001^a^
	*Regional hospitals*	269 (42.4)	73 (28.4)	
	*Local hospitals*	42 (6.6)	24 (9.3)	
**Insured residence areas**^**c**^				
	*Taipei D (36*.*6)*^d^	280 (44.4)	100 (39.2)	0.492^a^
	*Northern D (14*.*0)*^d^	65 (10.3)	30 (11.8)	
	*Central D (18*.*4)*^d^	143 (22.7)	70 (27.5)	
	*Southern D (13*.*9)*^d^	83 (13.2)	32 (12.5)	
	*Kaoping D (14*.*8)*^d^	46 (7.3)	15 (5.9)	
	*Eastern D (2*.*3)*^d^	14 (2.2)	8 (3.1)	

Data was shown in n (%).

RICI: recurrent infection or chronic inflammation.

UAO: tonsillar hypertrophy or upper airway obstruction.

Tumor: suspicious benign or malignant neoplasms.

D: district.

^a^Pearson’s chi-square test.

^b^Analysis of variance (ANOVA).

^c^missing n = 5.

^d^Percentage of the population in individual NHI division.

## Discussion

This report is the first study on the trends and indications of AT across all ages in Taiwan during 1997–2010. The overall incidence rate of AT was 14.7 per 100,000 population, and increased during 1997–2010. More than 99% of the study population received an AT on an inpatient basis. We noticed a low incidence of AT and a relatively small percentage of pediatric AT procedures in Taiwan. However, longitudinal data confirmed an increasing trend in the pediatric AT rates from 1997 to 2010. Regarding surgical indications for AT, although we reported a declining trend of infectious indications, treatment of RICI remained the most common cause of AT regardless of the calendar year, sex, age groups, hospital level, or insured residence areas. In the adult subgroup, we identified a decreasing prevalence of infectious indications, coincided with an increasing neoplastic indications. In the pediatric subgroup, the proportion of obstructive indications increased significantly. The logistic regression analyses indicated that after adjustment for confounders, the significant factors associated with the changing surgical indications for AT were the age in the adult subgroup and hospital level in the pediatric subgroup.

The overall incidence of AT varied from 1997 to 2010. The AT rate showed an increasing trend from 1997 to 2006, with the only exceptions in 2002 and 2003. During the severe acute respiratory syndrome (SARS) epidemic in 2003, elective surgeries were either canceled or postponed, and the AT rate dropped significantly in 2002 and 2003. After the SARS epidemic, the incidence of AT increased up to 185 cases in 2006. Major public health events such as SARS may affect the surgical rate, particularly for elective surgical procedures. However, the surgical rate declined again after 2006, with most of the reduction observed in adult UAO patients. Since 2000, an increasing amount of evidence has shown that solitary tonsillectomy as a treatment for adult SDB patients might have a limited effect, even if they present with grade IV (kissing) tonsils. Otolaryngologists usually recommend adult SDB patients to receive uvulopalatopharyngoplasty (UPPP), instead of solitary tonsillectomy, in those who do not tolerate to or are unwilling to use continuous positive airway pressure devices or other conservative treatment such as oral appliances [28]. In contrast, UAO has become a plausible indication for AT, especially in pediatric patients [3]. Because adenotonsillar hypertrophy is a major cause of obstructive sleep apnea (OSA) in children, AT is among the first-line therapies recommended for uncomplicated pediatric OSA [29, 30].

Two of the most common reasons for pediatric and young-adult AT are infection and UAO secondary to adenotonsillar hypertrophy [7, 31]. Although AT for a suspected or confirmed neoplasm is rarely performed in children, it is the third most prevalent indication for AT in adults [6]. The NHI has encouraged almost all patients with AT to undergo surgery on an inpatient basis. By contrast, ambulatory AT procedures in children have nearly doubled over the past decade in the United States, with only <3% of tonsillectomies performed in an inpatient setting [22]. In Taiwan, the inconvenience of admission may partly reduce the surgical rate in the pediatric population. One study found that even when physicians recommended a surgical procedure based on the current guidelines, parents often chose nonsurgical treatment options because of anecdotal reports or based on their own education or social experiences [26]. The percentage of surgical procedures in the <18 years subgroup (48.2%) is relatively small compared with the figures that have been reported by other studies [5, 16, 21, 31], reflecting parental concerns regarding AT in Taiwan.

The surgical indications for AT have fundamentally changed over time. Parker and Walner [3] demonstrated a shift in surgical indications for pediatric AT from infection toward obstruction. In the group aged <18 years, more than 85% underwent surgery due to a UAO. Koshy et al. [4] reported that incidence of OSA diagnosis among children aged < 4 years who underwent AT doubled between 2001 and 2011. Clement [32] presented an increase in the rate of ambulatory AT in children with conditions on the SDB spectrum from 26% in 2001 to 55% in 2011, whereas UAO became the most common indication for AT among preschool children. Our results confirmed an increasing trend toward surgical management for upper airway obstructive symptoms rather than for mainly infectious reasons. However, treatment of RICI remained the most common indication for AT across all age groups. Those results reveal a lack of awareness among pediatricians and otolaryngologists of the symptoms of SDB and the difficulty in diagnosing OSA in the pediatric population.

Tonsillectomy for tonsil asymmetry and suspicion of malignant diseases is rarely performed in children, but it is an indication for tonsillectomy in the adult population [33]. Compared with deep tonsil biopsies, tonsillectomies have a significantly higher probability of detecting occult tonsillar tumors [34]. In this study, the surgical indications for tonsillar neoplasm increased substantially from 1997 to 2010, especially after 2006. A possible reason for this trend was the Taiwanese government's implementation of free oral and pharyngeal mucosal screening among high-risk groups (those who aged 18 or above and have a history of smoking or betel nut chewing) from 2007. By the end of this National Cancer Prevention and Control project, 1440,000 high-risk candidates (aged 18 or above, with a history of smoking or betel nut chewing) had been screened. The screening rate was 28%. One thousand two hundred and forty-eight patients were diagnosed to have oral or oropharyngeal cancer during 2008–2009. The timing of this national policy correlated well with the increase in tumor tonsillectomy. Furthermore, increasing incidence rates of oropharyngeal cancers were discovered on a global scale [35].

In the distribution of surgical indications according to hospital level, we discovered an increasing trend in the proportion of RICI and a decreasing trend in the proportion of UAO and Tumor among local hospitals. This trend is also plausible because the diagnosis of SDB may require overnight polysomnography and sleep laboratory testing may not be available in local hospitals. Additionally, patients diagnosed with neoplasms preferred regional hospitals or medical centers to local hospitals. In the analysis of the surgical indications by insured residence area, we found higher-than-expected (based on the population in each division) numbers of ATs performed in the Taipei and Central districts. The surgical rate of AT in Taiwan seemed to be higher in the metropolitan areas than in rural areas. The abundance of medical resources in these metropolitan areas may be a contributing factor. However, insured residence area was not an independent factor associated with the distribution of the surgical indications for AT after adjustment for confounders.

This study had several limitations. First, the dataset did not include a chart review of the participants; therefore, detailed clinical data were not available. Second, because the inherent preference of coding could not be assessed from our results; the accuracy of the type of procedures performed and the surgical indications may have been affected. Third, this study included a homogeneous population of Chinese patients in Taiwan; the generalizability of our results to other ethnic populations may be limited. Finally, selection bias may still exist when using LHID2005 for trend analysis. Those patients who received surgery after 1997 but died before 2005 were not included in the LHID2005. The study population who became older after 2005 might have a higher risk of developing neoplasms. Moreover, the number of surgery in the pediatric population less than five years of age was underestimated between 2005 and 2010. All these biases were potential confounders in this study. Therefore, we also examined the LHID2000 and LHID2010, which concurred with our current finding, to substantiate our observed trends in LHID2001. However, a complete dataset from the entire population with an extended study period would allow for more accurate trend analysis and prediction.

## Conclusions

Epidemiological trends in AT have changed substantially. The annual incidence rate of AT shifted significantly due to major public health events. RICIs remained the most common surgical indication for AT across all ages during 1997–2010. Incidence of pediatric AT increased with a shift in surgical indications from infections to obstruction. In the adult, the proportion of AT performed for infections decreased, whereas those performed due to neoplasm increased significantly. The age in the adult subgroup and hospital level in the pediatric group were significant factors associated with the changing surgical indications for AT. These epidemiological data will add information for the daily practice of physicians and otolaryngologists, and contribute to the development of an evidence-based clinical guideline for these procedures.

## Supporting information

S1 File The trend in the adenotonsillectomy rate and the proportion of surgical indications by the calendar year. (DOCX)

S2 File Multinomial logistic regression contributors for the different surgical indications. (DOCX)
