# Supplementary material for: Factors associated with changing indications for adenotonsillectomy: A population-based longitudinal study
Source: PLoS One. 2018 May 29;13(5):e0193317. doi: 10.1371/journal.pone.0193317 (PMC5973846; doi:10.1371/journal.pone.0193317)
Supplement: S1 File — (DOCX) [file pone.0193317.s001.docx]

**Supporting Information**

**S1 File. The trend in the adenotonsillectomy rate and the proportion of surgical indications by the calendar year.**

Table 1a. The trend of adenotonsillectomy rate in three categories of surgical indications by gender

|  | | 1997 | 1998 | 1999 | 2000 | 2001 | 2002 | 2003 | 2004 | 2005 | 2006 | 2007 | 2008 | 2009 | 2010 | total |
| --- | --- | --- | --- | --- | --- | --- | --- | --- | --- | --- | --- | --- | --- | --- | --- | --- |
| female | UAO | 3 | 5 | 4 | 10 | 13 | 10 | 9 | 20 | 20 | 37 | 15 | 10 | 18 | 9 | 183 |
|  | RICI | 33 | 47 | 37 | 57 | 55 | 42 | 28 | 34 | 43 | 40 | 34 | 34 | 35 | 37 | 556 |
|  | Tumor | 1 | 2 | 2 | 2 | 1 | 1 | 3 | 2 | 1 | 2 | 9 | 5 | 6 | 5 | 42 |
| male | UAO | 10 | 12 | 15 | 15 | 31 | 39 | 37 | 39 | 41 | 48 | 23 | 26 | 26 | 20 | 382 |
|  | RICI | 52 | 42 | 40 | 38 | 57 | 47 | 31 | 37 | 42 | 54 | 54 | 52 | 51 | 32 | 629 |
|  | Tumor | 3 | 3 | 5 | 0 | 1 | 2 | 7 | 15 | 5 | 4 | 11 | 8 | 18 | 18 | 100 |

Table 1b. The trend of adenotonsillectomy rate in gender by three categories of surgical indications

|  | | 1997 | 1998 | 1999 | 2000 | 2001 | 2002 | 2003 | 2004 | 2005 | 2006 | 2007 | 2008 | 2009 | 2010 | total |
| --- | --- | --- | --- | --- | --- | --- | --- | --- | --- | --- | --- | --- | --- | --- | --- | --- |
| UAO | female | 3 | 5 | 4 | 10 | 13 | 10 | 9 | 20 | 20 | 37 | 15 | 10 | 18 | 9 | 183 |
|  | male | 10 | 12 | 15 | 15 | 31 | 39 | 37 | 39 | 41 | 48 | 23 | 26 | 26 | 20 | 382 |
| RICI | female | 33 | 47 | 37 | 57 | 55 | 42 | 28 | 34 | 43 | 40 | 34 | 34 | 35 | 37 | 556 |
|  | male | 52 | 42 | 40 | 38 | 57 | 47 | 31 | 37 | 42 | 54 | 54 | 52 | 51 | 32 | 629 |
| Tumor | female | 1 | 2 | 2 | 2 | 1 | 1 | 3 | 2 | 1 | 2 | 9 | 5 | 6 | 5 | 42 |
|  | male | 3 | 3 | 5 | 0 | 1 | 2 | 7 | 15 | 5 | 4 | 11 | 8 | 18 | 18 | 100 |

Figure 1a. The bar chart of adenotonsillectomy rate in the category of UAO by sex from 1997 to 2010 (*p* = 0.210)

Figure 1b. The bar chart of adenotonsillectomy rate in the category of RICI by sex from 1997 to 2010 (*p* = 0.144)

Figure 1c. The bar chart of adenotonsillectomy rate in the category of Tumor by sex from 1997 to 2010 (*p* = 0.504)

Table 2a. The trend of adenotonsillectomy rate in three categories of surgical indications by age groups

|  | | 1997 | 1998 | 1999 | 2000 | 2001 | 2002 | 2003 | 2004 | 2005 | 2006 | 2007 | 2008 | 2009 | 2010 | total |
| --- | --- | --- | --- | --- | --- | --- | --- | --- | --- | --- | --- | --- | --- | --- | --- | --- |
| UAO | <5 year | 0 | 4 | 6 | 3 | 1 | 2 | 4 | 6 | 4 | 7 | 7 | 4 | 2 | 1 | 51 |
|  | 5-11 year | 3 | 4 | 4 | 7 | 10 | 11 | 9 | 18 | 14 | 27 | 18 | 13 | 20 | 11 | 169 |
|  | 12-17 year | 0 | 0 | 3 | 0 | 3 | 1 | 1 | 1 | 3 | 5 | 3 | 4 | 6 | 7 | 37 |
|  | 18-40 year | 8 | 5 | 4 | 8 | 14 | 23 | 13 | 20 | 26 | 27 | 7 | 15 | 7 | 6 | 183 |
|  | >40 year | 2 | 4 | 2 | 7 | 16 | 12 | 19 | 14 | 14 | 19 | 3 | 0 | 9 | 4 | 125 |
| RICI | <5 year | 4 | 9 | 9 | 15 | 11 | 12 | 9 | 8 | 15 | 8 | 14 | 7 | 5 | 1 | 127 |
|  | 5-11 year | 25 | 20 | 21 | 26 | 45 | 33 | 20 | 24 | 25 | 39 | 25 | 34 | 30 | 28 | 395 |
|  | 12-17 year | 10 | 4 | 5 | 10 | 8 | 7 | 3 | 6 | 10 | 10 | 11 | 7 | 13 | 8 | 112 |
|  | 18-40 year | 34 | 39 | 30 | 29 | 37 | 27 | 18 | 27 | 26 | 25 | 23 | 24 | 24 | 16 | 379 |
|  | >40 year | 12 | 17 | 12 | 15 | 11 | 10 | 9 | 6 | 9 | 12 | 15 | 14 | 14 | 16 | 172 |
| Tumor | <5 year | 0 | 0 | 1 | 0 | 0 | 0 | 0 | 0 | 0 | 0 | 0 | 0 | 0 | 0 | 1 |
|  | 12-17 year | 1 | 0 | 0 | 0 | 0 | 0 | 0 | 0 | 0 | 0 | 0 | 1 | 0 | 1 | 3 |
|  | 18-40 year | 0 | 2 | 1 | 0 | 1 | 0 | 2 | 6 | 0 | 2 | 5 | 0 | 0 | 3 | 22 |
|  | >40 year | 3 | 3 | 5 | 2 | 1 | 3 | 8 | 11 | 6 | 4 | 15 | 12 | 24 | 19 | 116 |

Table 2b. The trend of adenotonsillectomy rate in age groups by three categories of surgical indications

|  | | 1997 | 1998 | 1999 | 2000 | 2001 | 2002 | 2003 | 2004 | 2005 | 2006 | 2007 | 2008 | 2009 | 2010 | total |
| --- | --- | --- | --- | --- | --- | --- | --- | --- | --- | --- | --- | --- | --- | --- | --- | --- |
| <5 year | UAO | 0 | 4 | 6 | 3 | 1 | 2 | 4 | 6 | 4 | 7 | 7 | 4 | 2 | 1 | 51 |
|  | RICI | 4 | 9 | 9 | 15 | 11 | 12 | 9 | 8 | 15 | 8 | 14 | 7 | 5 | 1 | 127 |
|  | Tumor | 0 | 0 | 1 | 0 | 0 | 0 | 0 | 0 | 0 | 0 | 0 | 0 | 0 | 0 | 1 |
| 5-11 year | UAO | 3 | 4 | 4 | 7 | 10 | 11 | 9 | 18 | 14 | 27 | 18 | 13 | 20 | 11 | 169 |
|  | RICI | 25 | 20 | 21 | 26 | 45 | 33 | 20 | 24 | 25 | 39 | 25 | 34 | 30 | 28 | 395 |
| 12-17 year | UAO | 0 | 0 | 3 | 0 | 3 | 1 | 1 | 1 | 3 | 5 | 3 | 4 | 6 | 7 | 37 |
|  | RICI | 10 | 4 | 5 | 10 | 8 | 7 | 3 | 6 | 10 | 10 | 11 | 7 | 13 | 8 | 112 |
|  | Tumor | 1 | 0 | 0 | 0 | 0 | 0 | 0 | 0 | 0 | 0 | 0 | 1 | 0 | 1 | 3 |
| 18-40 year | UAO | 8 | 5 | 4 | 8 | 14 | 23 | 13 | 20 | 26 | 27 | 7 | 15 | 7 | 6 | 183 |
|  | RICI | 34 | 39 | 30 | 29 | 37 | 27 | 18 | 27 | 26 | 25 | 23 | 24 | 24 | 16 | 379 |
|  | Tumor | 0 | 2 | 1 | 0 | 1 | 0 | 2 | 6 | 0 | 2 | 5 | 0 | 0 | 3 | 22 |
| >40 year | UAO | 2 | 4 | 2 | 7 | 16 | 12 | 19 | 14 | 14 | 19 | 3 | 0 | 9 | 4 | 125 |
|  | RICI | 12 | 17 | 12 | 15 | 11 | 10 | 9 | 6 | 9 | 12 | 15 | 14 | 14 | 16 | 172 |
|  | Tumor | 3 | 3 | 5 | 2 | 1 | 3 | 8 | 11 | 6 | 4 | 15 | 12 | 24 | 19 | 116 |

Figure 2a. The bar chart of adenotonsillectomy rate in the category of UAO by age groups from 1997 to 2010 (*p* < 0.001)

Figure 2b. The bar chart of adenotonsillectomy rate in the category of RICI by age groups from 1997 to 2010 (*p* = 0.068)

Figure 2c. The bar chart of adenotonsillectomy rate in the category of Tumor by age groups from 1997 to 2010 (*p* = 0.043)

Table 3a. The trend of adenotonsillectomy rate in three categories of surgical indications by hospital levels

|  | | 1997 | 1998 | 1999 | 2000 | 2001 | 2002 | 2003 | 2004 | 2005 | 2006 | 2007 | 2008 | 2009 | 2010 | total |
| --- | --- | --- | --- | --- | --- | --- | --- | --- | --- | --- | --- | --- | --- | --- | --- | --- |
| Medical center | UAO | 4 | 11 | 12 | 13 | 26 | 27 | 23 | 32 | 35 | 43 | 23 | 24 | 26 | 20 | 319 |
|  | RICI | 54 | 59 | 51 | 57 | 64 | 47 | 33 | 26 | 32 | 47 | 42 | 40 | 40 | 36 | 628 |
|  | Tumor | 3 | 2 | 4 | 0 | 1 | 1 | 8 | 12 | 4 | 4 | 9 | 7 | 14 | 11 | 80 |
| Regional Hospitals | UAO | 9 | 6 | 4 | 12 | 14 | 21 | 16 | 22 | 21 | 34 | 12 | 12 | 16 | 9 | 208 |
|  | RICI | 24 | 17 | 21 | 32 | 38 | 35 | 21 | 36 | 43 | 42 | 37 | 40 | 40 | 30 | 456 |
|  | Tumor | 0 | 2 | 3 | 2 | 1 | 2 | 2 | 5 | 2 | 2 | 11 | 6 | 9 | 11 | 58 |
| Local Hospitals | UAO | 0 | 0 | 3 | 0 | 4 | 1 | 7 | 5 | 5 | 8 | 3 | 0 | 2 | 0 | 38 |
|  | RICI | 7 | 13 | 5 | 6 | 10 | 7 | 5 | 9 | 10 | 5 | 9 | 6 | 6 | 3 | 101 |
|  | Tumor | 1 | 1 | 0 | 0 | 0 | 0 | 0 | 0 | 0 | 0 | 0 | 0 | 1 | 1 | 4 |

Table 2b. The trend of adenotonsillectomy rate in hospital levels by three categories of surgical indications

|  | | 1997 | 1998 | 1999 | 2000 | 2001 | 2002 | 2003 | 2004 | 2005 | 2006 | 2007 | 2008 | 2009 | 2010 | total |
| --- | --- | --- | --- | --- | --- | --- | --- | --- | --- | --- | --- | --- | --- | --- | --- | --- |
| UAO | M center | 4 | 11 | 12 | 13 | 26 | 27 | 23 | 32 | 35 | 43 | 23 | 24 | 26 | 20 | 319 |
|  | R hospitals | 9 | 6 | 4 | 12 | 14 | 21 | 16 | 22 | 21 | 34 | 12 | 12 | 16 | 9 | 208 |
|  | L hospitals | 0 | 0 | 3 | 0 | 4 | 1 | 7 | 5 | 5 | 8 | 3 | 0 | 2 | 0 | 38 |
| RICI | M center | 54 | 59 | 51 | 57 | 64 | 47 | 33 | 26 | 32 | 47 | 42 | 40 | 40 | 36 | 628 |
|  | R hospitals | 24 | 17 | 21 | 32 | 38 | 35 | 21 | 36 | 43 | 42 | 37 | 40 | 40 | 30 | 456 |
|  | L hospitals | 7 | 13 | 5 | 6 | 10 | 7 | 5 | 9 | 10 | 5 | 9 | 6 | 6 | 3 | 101 |
| Tumor | M center | 3 | 2 | 4 | 0 | 1 | 1 | 8 | 12 | 4 | 4 | 9 | 7 | 14 | 11 | 80 |
|  | R hospitals | 0 | 2 | 3 | 2 | 1 | 2 | 2 | 5 | 2 | 2 | 11 | 6 | 9 | 11 | 58 |
|  | L hospitals | 1 | 1 | 0 | 0 | 0 | 0 | 0 | 0 | 0 | 0 | 0 | 0 | 1 | 1 | 4 |

Figure 3a. The bar chart of adenotonsillectomy rate in the category of UAO by hospital levels from 1997 to 2010 (*p* = 0.216)

Figure 3b. The bar chart of adenotonsillectomy rate in the category of RICI by hospital levels from 1997 to 2010 (*p* = 0.001)

Figure 3c. The bar chart of adenotonsillectomy rate in the category of Tumor by hospital levels from 1997 to 2010 (*p* = 0.431)

Table 4a. The trend of adenotonsillectomy rate in three categories of surgical indications by insured residence areas

|  | | 1997 | 1998 | 1999 | 2000 | 2001 | 2002 | 2003 | 2004 | 2005 | 2006 | 2007 | 2008 | 2009 | 2010 | total |
| --- | --- | --- | --- | --- | --- | --- | --- | --- | --- | --- | --- | --- | --- | --- | --- | --- |
| Tapai D | UAO | 5 | 5 | 6 | 8 | 15 | 20 | 14 | 19 | 20 | 31 | 16 | 17 | 20 | 11 | 207 |
|  | RICI | 39 | 47 | 37 | 44 | 49 | 34 | 24 | 33 | 37 | 45 | 38 | 39 | 39 | 24 | 529 |
|  | Tumor | 2 | 2 | 4 | 1 | 2 | 1 | 8 | 10 | 4 | 2 | 7 | 4 | 8 | 9 | 64 |
| Northern D | UAO | 1 | 1 | 5 | 2 | 5 | 6 | 5 | 4 | 7 | 6 | 3 | 5 | 3 | 5 | 58 |
|  | RICI | 11 | 9 | 7 | 9 | 13 | 12 | 12 | 9 | 11 | 12 | 3 | 7 | 4 | 10 | 129 |
|  | Tumor | 2 | 1 | 1 | 0 | 0 | 0 | 1 | 0 | 0 | 1 | 1 | 2 | 3 | 5 | 17 |
| Central D | UAO | 6 | 7 | 3 | 9 | 12 | 12 | 16 | 11 | 13 | 19 | 6 | 6 | 8 | 4 | 132 |
|  | RICI | 12 | 9 | 10 | 21 | 19 | 22 | 9 | 14 | 22 | 22 | 19 | 19 | 15 | 16 | 229 |
|  | Tumor | 0 | 0 | 0 | 1 | 0 | 1 | 0 | 3 | 1 | 2 | 3 | 2 | 6 | 1 | 20 |
| Southern D | UAO | 0 | 1 | 4 | 1 | 4 | 7 | 9 | 7 | 13 | 17 | 6 | 5 | 7 | 5 | 86 |
|  | RICI | 9 | 8 | 8 | 8 | 11 | 11 | 5 | 10 | 9 | 9 | 13 | 13 | 9 | 8 | 131 |
|  | Tumor | 0 | 0 | 1 | 0 | 0 | 1 | 0 | 1 | 1 | 0 | 6 | 3 | 3 | 4 | 20 |
| Kaoping D | UAO | 0 | 2 | 1 | 3 | 7 | 4 | 1 | 9 | 8 | 11 | 5 | 2 | 6 | 1 | 60 |
|  | RICI | 13 | 9 | 12 | 10 | 15 | 9 | 3 | 3 | 3 | 3 | 8 | 5 | 14 | 7 | 114 |
|  | Tumor | 0 | 2 | 0 | 0 | 0 | 0 | 1 | 2 | 0 | 1 | 3 | 2 | 4 | 4 | 19 |
| Eastern D | UAO | 1 | 1 | 0 | 2 | 1 | 0 | 1 | 3 | 0 | 1 | 1 | 1 | 0 | 3 | 15 |
|  | RICI | 1 | 4 | 3 | 2 | 4 | 1 | 2 | 0 | 3 | 2 | 3 | 0 | 3 | 3 | 31 |
|  | Tumor | 0 | 0 | 1 | 0 | 0 | 0 | 0 | 1 | 0 | 0 | 0 | 0 | 0 | 0 | 2 |

Table 4b. The trend of adenotonsillectomy rate in insured residence areas by three categories of surgical indications

|  | | 1997 | 1998 | 1999 | 2000 | 2001 | 2002 | 2003 | 2004 | 2005 | 2006 | 2007 | 2008 | 2009 | 2010 | total |
| --- | --- | --- | --- | --- | --- | --- | --- | --- | --- | --- | --- | --- | --- | --- | --- | --- |
| UAO | Taipei D | 5 | 5 | 6 | 8 | 15 | 20 | 14 | 19 | 20 | 31 | 16 | 17 | 20 | 11 | 207 |
|  | Northern D | 1 | 1 | 5 | 2 | 5 | 6 | 5 | 4 | 7 | 6 | 3 | 5 | 3 | 5 | 58 |
|  | Central D | 6 | 7 | 3 | 9 | 12 | 12 | 16 | 11 | 13 | 19 | 6 | 6 | 8 | 4 | 132 |
|  | Southern D | 0 | 1 | 4 | 1 | 4 | 7 | 9 | 7 | 13 | 17 | 6 | 5 | 7 | 5 | 86 |
|  | Kaoping D | 0 | 2 | 1 | 3 | 7 | 4 | 1 | 9 | 8 | 11 | 5 | 2 | 6 | 1 | 60 |
|  | Eastern D | 1 | 1 | 0 | 2 | 1 | 0 | 1 | 3 | 0 | 1 | 1 | 1 | 0 | 3 | 15 |
| RICI | Taipei D | 39 | 47 | 37 | 44 | 49 | 34 | 24 | 33 | 37 | 45 | 38 | 39 | 39 | 24 | 529 |
|  | Northern D | 11 | 9 | 7 | 9 | 13 | 12 | 12 | 9 | 11 | 12 | 3 | 7 | 4 | 10 | 129 |
|  | Central D | 12 | 9 | 10 | 21 | 19 | 22 | 9 | 14 | 22 | 22 | 19 | 19 | 15 | 16 | 229 |
|  | Southern D | 9 | 8 | 8 | 8 | 11 | 11 | 5 | 10 | 9 | 9 | 13 | 13 | 9 | 8 | 131 |
|  | Kaoping D | 13 | 9 | 12 | 10 | 15 | 9 | 3 | 3 | 3 | 3 | 8 | 5 | 14 | 7 | 114 |
|  | Eastern D | 1 | 4 | 3 | 2 | 4 | 1 | 2 | 0 | 3 | 2 | 3 | 0 | 3 | 3 | 31 |
| Tumor | Taipei D | 2 | 2 | 4 | 1 | 2 | 1 | 8 | 10 | 4 | 2 | 7 | 4 | 8 | 9 | 64 |
|  | Northern D | 2 | 1 | 1 | 0 | 0 | 0 | 1 | 0 | 0 | 1 | 1 | 2 | 3 | 5 | 17 |
|  | Central D | 0 | 0 | 0 | 1 | 0 | 1 | 0 | 3 | 1 | 2 | 3 | 2 | 6 | 1 | 20 |
|  | Southern D | 0 | 0 | 1 | 0 | 0 | 1 | 0 | 1 | 1 | 0 | 6 | 3 | 3 | 4 | 20 |
|  | Kaoping D | 0 | 2 | 0 | 0 | 0 | 0 | 1 | 2 | 0 | 1 | 3 | 2 | 4 | 4 | 19 |
|  | Eastern D | 0 | 0 | 1 | 0 | 0 | 0 | 0 | 1 | 0 | 0 | 0 | 0 | 0 | 0 | 2 |

Figure 4a. The bar chart of adenotonsillectomy rate in the category of UAO by insured residence areas from 1997 to 2010 (*p* = 0.412)

Figure 4b. The bar chart of adenotonsillectomy rate in the category of RICI by insured residence areas from 1997 to 2010 (*p* = 0.268)

Figure 4c. The bar chart of adenotonsillectomy rate in the category of Tumor by insured residence areas from 1997 to 2010 (*p* = 0.630)
