# Supplementary material for: Factors associated with changing indications for adenotonsillectomy: A population-based longitudinal study
Source: PLoS One. 2018 May 29;13(5):e0193317. doi: 10.1371/journal.pone.0193317 (PMC5973846; doi:10.1371/journal.pone.0193317)
Supplement: S2 File — (DOCX) [file pone.0193317.s002.docx]

**Supporting Information**

**S2 File. Multinomial logistic regression contributors for the different surgical indications.**

**Table A. Multinomial logistic regression contributors for the different surgical indications in all subjects.** The results showed that age was the only significant factor associated with the different surgical indications. The effect of sex, an insured residence area and hospital levels were not statistically significant after all the variables were controlled.

|  | | Adjusted OR | | 95% CI | *p*-Value |
| --- | --- | --- | --- | --- | --- |
| RICI | *constant* |  | |  | 0.286 |
|  | *Female* | 1.961 | | (1.579–2.436) | <0.001 |
|  | *<5y (n=179)* | 1.980 | | (1.317–2.977) | 0.001 |
|  | *5-18y (n=733)* | 1.969 | | (1.473–2.632) | <0.001 |
|  | *19-29y (n=323)* | 1.715 | | (1.212–2.427) | 0.002 |
|  | *30-39y (n=244)* | 1.223 | | (0.852–1.756) | 0.275 |
|  | *≧40y (n=413)* |  | |  |  |
|  | *Taipei D* | 1.411 | | (0.737–2.703) | 0.299 |
|  | *Northern D* | 1.213 | | (0.600–2.453) | 0.590 |
|  | *Central D* | 0.923 | | (0.474–1.795) | 0.813 |
|  | *Southern D* | 0.812 | | (0.408–1.616) | 0.553 |
|  | *Kaoping D* | 1.096 | | (0.540–2.223) | 0.800 |
|  | *Eastern D* |  | |  |  |
|  | *Medical centers* | 0.675 | | (0.447–1.021) | 0.062 |
|  | *Regional hospitals* | 0.787 | | (0.515–1.203) | 0.268 |
|  | *Local hospitals* |  | |  |  |
| Tumor | *constant* | |  |  | 0.203 |
|  | *Female* | | 0.723 | (0.469–1.117) | 0.144 |
|  | *<5y (n=179)* | | 0.019 | (0.003–0.143) | <0.001 |
|  | *5-18y (n=733)* | | 0.020 | (0.007–0.055) | <0.001 |
|  | *19-29y (n=323)* | | 0.072 | (0.030–0.173) | <0.001 |
|  | *30-39y (n=244)* | | 0.172 | (0.093–0.316) | <0.001 |
|  | *≧40y (n=413)* | |  |  |  |
|  | *Taipei D* | | 1.540 | (0.311–7.625) | 0.597 |
|  | *Northern D* | | 1.708 | (0.319–9.157) | 0.532 |
|  | *Central D* | | 0.709 | (0.136–3.704) | 0.683 |
|  | *Southern D* | | 0.974 | (0.184–5.165) | 0.976 |
|  | *Kaoping D* | | 1.205 | (0.227–6.388) | 0.826 |
|  | *Eastern D* | |  |  |  |
|  | *Medical centers* | | 2.258 | (0.747–6.825) | 0.149 |
|  | *Regional hospitals* | | 3.012 | (0.974–9.311) | 0.056 |
|  | *Local hospitals* | |  |  |  |

OR=odds ratio; CI=confidence interval

**Table B. Multinomial logistic regression contributors for the different surgical indications in adult group (****≥18 y/o).** The results showed that age was the only significant factor associated with the different surgical indications (RICI vs. UAO, Tumor vs. UAO) in the adult subgroup. Compared with RICI, UAO occurred more frequently in the higher hospital level. The effect of sex, an insured residence area, and hospital levels was not statistically significant in the Tumor group (vs. UAO) after all the variables were controlled.

|  | | Adjusted OR | | 95% CI | *p*-Value |
| --- | --- | --- | --- | --- | --- |
| RICI | *constant* |  | |  | 0.143 |
|  | *Female* | 3.199 | | (2.294–4.241) | <0.001 |
|  | *18-40y* | 1.554 | | (1.136–2.126) | 0.006 |
|  | *>40y* |  | |  |  |
|  | *Taipei D* | 1.152 | | (0.446–2.978) | 0.770 |
|  | *Northern D* | 1.056 | | (0.377–2.955) | 0.918 |
|  | *Central D* | 0.818 | | (0.306–2.185) | 0.688 |
|  | *Southern D* | 0.541 | | (0.197–1.486) | 0.233 |
|  | *Kaoping D* | 0.714 | | (0.261–1.947) | 0.510 |
|  | *Eastern D* |  | |  |  |
|  | *Medical centers* | 0.404 | | (0.447–1.021) | 0.008 |
|  | *Regional hospitals* | 0.285 | | (0.515–1.203) | <0.001 |
|  | *Local hospitals* |  | |  |  |
| Tumor | *constant* | |  |  | 0.490 |
|  | *Female* | | 0.827 | (0.519–1.316) | 0.423 |
|  | *18-40y* | | 0.124 | (0.074–0.210) | <0.001 |
|  | *>40y* | |  |  |  |
|  | *Taipei D* | | 1.341 | (0.249–7.212) | 0.733 |
|  | *Northern D* | | 1.527 | (0.259–9.011) | 0.640 |
|  | *Central D* | | 0.619 | (0.109–3.527) | 0.589 |
|  | *Southern D* | | 0.821 | (0.143–4.721) | 0.825 |
|  | *Kaoping D* | | 0.949 | (0.166–5.441) | 0.953 |
|  | *Eastern D* | |  |  |  |
|  | *Medical centers* | | 2.009 | (0.535–7.546) | 0.302 |
|  | *Regional hospitals* | | 2.087 | (0.545–7.996) | 0.283 |
|  | *Local hospitals* | |  |  |  |

OR=odds ratio; CI=confidence interval

**Table C. Logistic regression contributors for the different surgical indications in pediatric group (<18 y/o).** The results showed that hospital levels were the only significant factor associated with the different surgical indications in the pediatric subgroup. The effect of age, sex, and an insured residence area was not statistically significant after all the variables were controlled.

(UAO=0, RICI=1)

|  | Adjusted OR | 95% CI | *p*-Value |
| --- | --- | --- | --- |
| *constant* |  |  | 0.926 |
| *Female* | 1.241 | (0.909–1.693) | 0.174 |
| *Age* | 0.998 | (0.959–1.038) | 0.910 |
| *Taipei D* |  |  | 0.456 |
| *Northern D* | 1.875 | (0.750–4.689) | 0.179 |
| *Central D* | 1.488 | (0.553–3.999) | 0.431 |
| *Southern D* | 1.397 | (0.549–3.551) | 0.483 |
| *Kaoping D* | 1.527 | (0.575–4.054) | 0.396 |
| *Eastern D* | 2.123 | (0.731–6.166) | 0.166 |
| *Medical centers* |  |  | <0.001 |
| *Regional hospitals* | 1.089 | (0.630–1.884) | 0.760 |
| *Local hospitals* | 2.055 | (1.162–3.634) | 0.013 |

OR=odds ratio; CI=confidence interval
